# Study of the Spanish Society of Medical Oncology (SEOM) on the access to oncology drugs and predictive biomarkers in Spain

**DOI:** 10.1007/s12094-020-02366-y

**Published:** 2020-06-12

**Authors:** A. Rodríguez-Lescure, F. A. de la Peña, E. Aranda, A. Calvo, E. Felip, P. Garrido, R. Vera

**Affiliations:** 1 Medical Oncology Department, Hospital General Universitario de Elche, Elche, Spain; 2 Haematology and Medical Oncology Department, Hospital Universitario Morales Meseguer, Murcia, Spain; 3 Oncology Department, Maimonides Institute of Biomedical Research (IMIBIC), Reina Sofía Hospital, University of Córdoba, Spanish Cancer Network (RTICC), Instituto de Salud Carlos III, Madrid, Spain; 4 Medical Oncology Department, Hospital General Universitario Gregorio Marañón, Madrid, Spain; 5 Medical Oncology Department, Hospital Universitario Vall D’Hebron, Barcelona, Spain; 6 Medical Oncology Department, Hospital Universitario Ramón Y Cajal, Madrid, Spain; 7 Medical Oncology Department, Complejo Hospitalario de Navarra, Navarrabiomed, IdISNA, Pamplona, Spain

**Keywords:** Oncology, Drug, Biomarker, Equity, Access, Barriers, Spain

## Abstract

**Purpose:**

The Spanish Society of Medical Oncology (SEOM) has carried out a study to analyse the conditions of access to oncology drugs in clinical practice in Spain. For the first time, the access of predictive biomarkers has also been analyzed.

**Methods:**

A questionnaire was sent to 146 hospitals in Spain to collect information on the process of approval of 11 oncology drugs of an unquestionable clinical benefit and five predictive biomarkers of mandatory determination for specific treatments.

**Results:**

Results highlight the still existing differences in the access of oncology drugs, as well as the newly identified differences in the access to predictive biomarkers between Autonomous Communities (AACC) in Spain, as well as between different hospitals within the same Autonomous Community.

Conclusions

The SEOM considers it necessary to reduce the differences identified, increase homogeneity, and improve conditions of access to oncology drugs and biomarkers, and makes proposals to address these issues.

## Introduction

Cancer is a primary health problem due to its high incidence, prevalence, and mortality rates. Worldwide in 2018, 18.1 million new cases cancer were identified and a total of 9.5 million cancer-associated deaths were registered. In the next 20 years, the incidence of cancer is predicted to increase by approximately 64% and to reach an estimate of 29.5 million new cases in 2040 [1].

In Spain, an estimated total of 227,234 new cancer cases were detected in 2019 [1], being the second leading cause of mortality in the overall population following cardiovascular disease, and the leading overall cause in the male population [2].

Nevertheless, the incidence and mortality trends in some cancers are starting to level off, with some types of cancers even beginning to show decreasing rates. This suggests an increase in the efficacy of new therapeutic approaches as well as preventative policies, both at primary and secondary levels [2]. In the past years, cancer survival rates have increased steadily in Europe, especially in some cancer types. In Spain, survival rates are on average 53% at 5 years, which is comparable to those of similar countries [3–5].

One of the main developments in the treatment of patients with cancer is the possibility of making therapeutic decisions based on the genomic and molecular characteristics of each patient’s tumor. This approach is known as precision medicine and it allows patients to receive personalized treatments, with a greater efficacy and a lower toxicity than the conventional treatments [6, 7].

Advances in precision medicine go hand in hand with the identification and determination of new biomarkers. Nevertheless, many biomarkers are still not available in day-to-day clinical practice due to the numerous barriers and threats that limit their accessibility [6, 7].

An study on access to novel drugs for NSCLC carried out in Central and Southeastern Europe (CEE) concluded that the access to novel drugs was suboptimal in CEE countries and that availability of drugs was not in accordance with their value scores, like the ESMO Magnitude of Clinical Benefit Scale [8].

In Spain, after EU authorization, oncologic drugs must first be approved by the Spanish Agency of Medicines and Medical Products (AEMPS) and, after that, the Therapeutic Positioning Report (IPT) is issued. This report serves as a reference for any action related to the acquisition and promotion of the rational use of the drug. The Inter-Ministerial Commission for the Pricing of Medicines (CIPM) is then responsable for establishing the Pricing and Reimbursement (PR) within the Spanish Public Health System (SNS) [9, 10]. Afterwards, Autonomous Communities (AACC) will cover the expense of the drugs as part of their regional budgets.

In this context, following the results obtained from the 2015 study on the access of pharmaceutical drugs in Spain, the Spanish Society of Medical Oncology (SEOM) has carried out the present study to evaluate and follow up, 4 years later, the access conditions to oncology drugs in real clinical practice throughout the Spanish territory. Furthermore, for the first time, the access conditions in clinical practice to predictive biomarkers mandatory by Data Sheet as a means of selecting patients for some specific oncology drugs have also been analyzed.

The aim of this study is to identify the different decision-making mechanisms involved in the approval and access to oncology drugs and predictive biomarkers in clinical practice in Spain, analyse the availability of approved drugs and predictive biomarkers, as well as compare, where possible, how the situation has changed since 2015, and make new proposals on ways of tackling the barriers and inequalities identified.

## Materials and methods

An electronic questionnaire was distributed via email to the Medical Oncology Services of 146 hospitals in Spain, covering all 17 AACC and the Autonomous City of Ceuta. It was elaborated by a group of medical oncologists belonging to the SEOM, who also acted as the Panel of Experts (PE) and are the authors of this article. They took part in work meetings throughout the project to monitor and evaluate results, identify areas of improvement, and make subsequent proposals.

The PE decided the drugs and biomarkers included in this study based on the following criteria: (1) unquestionable clinical benefit, (2) funding granted in Spain between 2016 and 2018, (3) drugs whose access issues could have a great effect on the adequate treatment of patients, and (4) could be representative of current access conditions in Spain. As a result, the PE selected approved indications for 11 drugs and five predictive biomarkers mandatory for the prescription of specific oncology drugs under Data Sheet requirements, to address the following conditions: non-microcytic lung cancer (drugs: pembrolizumab, atezolizumab, and nivolumab; biomarkers: PDL-1, ALK, and ROS-1), breast cancer (drugs: ribociclib and palbociclib), melanoma (drugs: dabrafenib + trametinib and cobimetinib + vemurafenib; biomarker: BRAF), and ovarian cancer (drug: olaparib; biomarker: BRCA1/BRCA2) (Tables 1 and 2).

**Table 1:** Description of the drugs and indications analysed in this study

Lung cancer	Pembrolizumab	1. Monotherapy for the first-line treatment of metastatic Non-Small Cell Lung Carcinoma (NSCLC) in adults with tumours that express PD-L1 with a tumour maker proportion (TMP) ≥ 50% without positive tumour mutations o the EGFR or ALK genes 2. Monotherapy for the treatment of locally advanced or metastatic NSCLC in adults with tumours that express PD-L1 with a TPS ≥ 1% and that have received at least one previous chemotherapy treatment
	Atezolizumab	Monotherapy for the treatment of adult patients with locally advanced or metastatic Non-Small Cell Lung Carcinoma (NSCLC) that have received prior treatment with chemotherapy
	Nivolumab	Monotherapy for the treatment of adult patients with locally advanced or metastatic Non-Small Cell Lung Carcinoma (NSCLC) that have received prior treatment with chemotherapy
Breast cancer	Ribociclib	In combination with an aromatase inhibitor as an initial hormonal treatment of post-menopausal women with locally advanced or metastatic breast cancer, positive for the Hormonal Receptor (HR), and negative for the Human Epidermal Growth Factor 2 (HER2)
	Palbociclib	Treatment of metastatic or locally advanced Hormonal Receptor (HR) positive and Human Epidermal Growth Factor Receptor 2 (HER2) negative breast cancer
Melanoma	Dabrafenib y Trametinib	Trametinib in combination dabrafenib for the treatment of adult patients with non-resectable or metastatic melanoma with a BRAF 600 mutation
	Cobimetinib y Vemurafenib	Cobimetinib in combination with vemurafenib for the treatment of adult patients with non-resectable or metastatic melanoma with a BRAF V600 mutation
Ovarian cancer	OLAPARIB	Monotherapy for the treatment of adult patients with BRCA-mutated (germline or somatic) advanced high-grade serous epithelial ovarian cancer, fallopian tube or primary peritoneal cancer, in relapse and sensitive to platinum, following first-line platinum-based chemotherapy (complete or partial response)

**Table 2:** Description of the biomarkers and indications analysed in this study

Lung cancer	PD-L1	Expression of the PD-L1 predictive biomarker in response to the treatment of Non-Small-Cell Lung Carcinoma (NSCLC)
	ALK	Rearrangement of ALK as a predictive biomarker in response to the treatment of NSCLC
	ROS-1	Translocation of ROS-1 as a predictive biomarker in response to the treatment of NSCLC
Melanoma	BRAF	Mutation of BRAF as a predictive biomarker in response to the treatment of Melanoma
Ovarian cancer	BRCA1/BCRA2	Mutation in the BRCA1/BRCA2 genes as predictive biomarkers in response to the treatment of Ovarian Cancer

The questionnaire requested descriptive data of each centre and information on the commissions and decision-making bodies involved in the access. For each drug/indication, information requested included dates of application and approval/denial for use; approved usage conditions and any barriers or limitations. For each biomarker, information requested included availability of procedures for requesting their determination; in house or outsourced determination and response times; usage criteria; funding of the determination in clinical practice; management and registering platforms, and any barriers or limitations identified. The detail of the questions included is shown in Annexes 1, 2, and 3.

The time taken from the EC authorization and from the fixing of PR conditions in Spain until the approval of each drug /indication for its prescription in each individual hospital was measured. The minimum, maximum, and median times were also calculated.

A literature review was carried out on all relevant content published in the web pages of the EC, EMA, General Directorate of Pharmacy and Medicinal Products of the Spanish Ministry of Health, the AEMPS, regional health services as well as the Official Gazettes of each Autonomous Community, regarding the approval and use of biomarkers, as well as the access to oncology drugs.

This study started in December 2018 and ended in May 2019.

## Results

The questionnaire was completed by 84 hospitals (58% of the centres contacted). Of these, 80 are public hospitals and four are private hospitals, one of these being an associated private centre. The participating centres represent all 17 AACC as well as the Autonomous City of Ceuta. According to the responses, 62% of the centres are of level 3 complexity, 29% are of level 2 complexity, and 9% are of level 1 complexity; 56% of the participant centres correspond to areas of over 300,000 inhabitants, 22% of areas between 200,000 and 300,000 inhabitants, and 22% to areas below 200,000 inhabitants.

## Oncology drugs

### Commissions and decision-making bodies that affect the access

According to the responses, in 51% of cases, decisions regarding the access of the drugs are taken at hospital/health Area; in 42% of cases, decisions are made at Autonomous Community level; and in 5% of cases, decisions are made at a combination of levels. Results show there are clear variations between AACC. In Asturias, Baleares, Cantabria, C. Valenciana, Galicia, La Rioja, Navarra, and País Vasco, decisions are always made at Autonomous Community level; whereas in Castilla La Mancha and Madrid, decisions are always made at a hospital/health Area level. In the remaining AACC, responses obtained vary between hospitals.

No information was provided on the commissions in 58% of the answers, either because it is not publically available (31%) or because it is unknown (27%).

In cases where participants have information about the decision-making commission, they have indicated that at least one medical oncologist participates in it.

### Conditions for the use and the need for additional approvals

A total of 60% of the responses indicate that, despite having been authorised by the AEMPS and having fixed PR conditions, the use of the drugs requires an additional authorization by the Hospital´s Pharmacotherapy Commission (HPC).

A total of 20% of responses indicate that at least one of the drugs/indications included in this study had not been approved (15%) or were pending approval (5%). It must be noted that this questionnaire was carried out during the first 4 months of 2019 and that the drugs/indications included in the study were designated PR conditions between January 2016 and April 2018.

There are differences in the responses obtained regarding the criteria which govern the use of the drugs. These criteria are specified by, amongst others, regional reports, hospital reports developed by the HPC, and positioning reports of the SEOM. Furthermore, the criteria established for their use are not always the same as those established for their commercialization in Spain.

### Analysis of authorization times for the approval of prescriptions (Table 3)

**Table 3:** Time taken in months from the PR fixation of an indication until the approval for its use

Autonomous community	Pembrolizumab 1L				Pembrolizumab 2L				Atezolizumab				Nivolumab				Ribociclib			
	Med	Min	Max	N	Med	Min	Max	N	Med	Min	Max	N	Med	Min	Max	N	Med	Min	Max	N
Andalucía	12	8	16	12	11	1	14	11	4	1	8	12	20	3	36	12	6.5	2	13	10
Aragón	–	–	–	–	–	–	–	–	–	–	–	–	–	–	–	–	4	4	4	–
Asturias	10	10	10	1	–	–	–	–	0	0	0	1	–	–	–	–	–	–	–	–
Baleares	10.5	6	15	2	17.5	13	22	2	7	7	7	2	24	24	24	2	5	5	5	2
C. de Madrid	9	1	14	9	13	1	18	9	7	3	8	9	20.5	1	29	9	4	3	12	7
C. La Mancha	15	15	15	5	22	22	26	6	7	7	7	6	12	10	13	6	–	–	–	5
C. Valenciana	13	13	13	6	9.5	3	16	6	5	5	5	6	8	4	12	6	3,5	2	5	6
Canarias	–	–	–	–	–	–	–	–	–	–	–	–	–	–	–	–	–	–	–	–
Cantabria	0	0	0	1	–	–	–	–	–	–	–	–	19	19	19	1	–	–	–	
Castilla y León	6	6	6	4	10	10	10	4	8	8	8	4	14	13	17	5	–	–	–	–
Cataluña	8	5	9	11	13.5	0	16	11	2	0	16	9	15	2	26	10	7	4	9	10
Ceuta	13	13	13	1	–	–	–	–	–	–	–	–	33	33	33	1	–	–	–	
Extremadura	–	–	–	–	––	–	–	–	–	–	–	–	1	1	1	1	–	–	–	–
Galicia	18	18	18	4	–	–	–	–	3	3	3	4	–	–	–	–	–	–	–	–
La Rioja	0	0	0	1	–	–	–	–	6	6	6	1	–	–	–	–	–	–	–	–
Murcia	15	15	15	2	1	1	1	2	4	4	4	2	13	10	16	2	7	7	7	2
Navarra	9	9	9	1	5	5	5	1	7	7	7	1	–	–	–	–	–	–	–	–
País Vasco	3.5	3	4	3	11	11	11	3	8	8	8	2	23	23	23	2	6.5	6	7	3
Global Median (months)	10				11				6				17				6			
	Pembrolizumab 1L				Pembrolizumab 2L				Atezolizumab				Nivolumab				Ribociclib			
Date of approval by the EC	27/01/2017				29/07/2016				21/09/2017				28/10/2015				22/08/2017			
Date of PR fixing for the indication	01/08/2017				01/01/2017				01/04/2018				01/02/2016				01/11/2017			

Autonomous community	Palbocilib				Dabrafenib and trametinib				Cobimetinib and vemurafenib				Olaparib			
	Med	Min	Max	*N*	Med	Min	Max	*N*	Med	Min	Max	*N*	Med	Min	Max	*N*
Andalucía	7.5	5	11	11	9	5	22	11	6	0	16	11	28	14	35	10
Aragón	4	4	4	3	–	–	–	–	–	–	–	–	–	–	–	3
Asturias	0	0	0	1	11	11	11	1	5	5	5	1	13	13	13	1
Baleares	5	5	5	2	–	–	–	–	–	–	–	–	29	29	29	2
C. de Madrid	4	3	10	10	19	14	25	8	16	16	19	7	13	1	33	6
C. La Mancha	11	1	12	6	9	9	9	5	7	3	11	5	–	–	–	4
C. Valenciana	2	2	2	7	14	14	14	5	12	12	12	5	0	0	0	6
Canarias	–	–	–	–	10	10	10	1	3	3	3	1	32	32	32	1
Cantabria	–	–	–	–	21	21	21	1	21	21	21	1	–	–	–	1
Castilla y León	7	7	7	4	18	18	18	4	12	12	12	5	20	20	20	3
Cataluña	5.5	2	8	9	9.5	0	30	6	16	8	24	6	3	0	5	10
Ceuta	–	–	–	–	–	–	–	–	–	–	–	–	–	–	–	
Extremadura	–	–	–	–	–	–	–	–	–	–	–	–	–	–	–	1
Galicia	2.5	1	4	5	9	9	9	4	3	3	3	4	23	23	23	4
La Rioja	11	11	11	1	-	–	–	–	–	–	–	-	12	12	12	1
Murcia	2	2	2	2	0	0	0	2	2	2	2	2	1	1	1	2
Navarra	4	4	4	1	18	18	18	1	12	12	12	1	10	10	10	1
País Vasco	6	6	7	3	6	5	15	3	5	0	10	3	1	1	1	2
Global median (months)	5				10				7				13			
	Palbocilib				Dabrafenib and Trametinib				Cobimetinib and Vemurafenib				Olaparib			
Date of approval by the EC	09/11/2016				n/a				n/a				16/12/2014			
Date of PR fixing for the indication	01/11/2017				01/04/2016				01/10/2016				01/01/2016			

*Med* median, *Min* minimum, *Max* maximum, *N* number of centres that have answered

According to the responses, the time taken from the designation of PR until approval for prescription within the different hospitals shows great variability, ranging between 0 and 36 months. For palbociclib, the median was of 5 months, (ranging from 1 to 12 months); 5.75 months for ribociclib (2–13 months); 6.5 months for atezolizumab in NSCLC (1–16 months); 7 months for cobimetinib + vemurafenib (0 to 24 months); 10 months for dabrafenib + trametinib (5–30 months); 10.5 months for pembrolizumab 1st L NSCLC (1 to 18 months); 11 months for pembrolizumab in 2ndL NSCLC (1–26 months); 13 months for olaparib (1–35 months); and 17 months for nivolumab in NSCLC (1 to 36 months).

Timescales from the EC authorization until the approval for prescription at different hospitals ascend up to 48 months (olaparib), with medians ranging between 87.5 months (ribociclib between 5 and 18 months) and 26 months (olaparib between 14 and 18 months). For the remaining drugs/indications, median timescales in ascending order are the following: 13 months for atezolizumab (7–23 months); 16.5 months for palbociclib (12–24 months); 17 months for pembrolizumab 1st NSCLC (7–25 months), 17 months for pembrolizumab 2nd NSCLC (7–32 months), and 21 months for nivolumab (5–40 months).

Table 3 shows the median time from the designation of the PR until the authorization at each Autonomous Community, as well as the minimum, maximum, and global median for each of the drugs/indication. The time taken from the EC authorization until PR is also shown.

### Barriers to the access of oncology drugs

In 43% of the centres, barriers to the access and use have been detected for at least one of the drugs analyzed. These barriers primarily consist of: delays and slowness of the approval process; requirement of a supporting report for each patient, which means additional delays and can result in the denial of the request; use criteria more restrictive than those established for the drug commercialization in Spain; selection of other drugs with the same indication and a different mechanism of action for use in the centre; non-approval or a pending approval of the drug in the centre.

## Biomarkers

### Commissions and decision-making bodies

Responses to the questionnaire and the bibliographic review show that there are no standardized criteria for the implementation of biomarkers in clinical practice in Spain.

According to the answers, in most AACC, there are great variations between hospitals regarding how and where the decisions for the approval of biomarkers are made.

In 84% of centres, there is no established commission or decision-making body responsible for making decisions on the access of predictive biomarkers or there is no information in this regard.

In the majority of centres that do have a decision-making body, such decisions are made in one of the following: Tumour or Molecular Pathology Committee; Tumours or Genetic Committee.

Some participants have indicated that, at the end date of the study (May 2019), some of the AACC were implementing, or starting to work on the implementation of mechanisms for the management of biomarkers determination in clinical practice and on their possible inclusion in the portfolio of services offered by the region.

### Access to biomarkers

In 96% of the participating centres, it is possible to request the determination of the predictive biomarkers included in this study (93–98% depending on the biomarker).

There is great diversity about the criteria followed for the access. In 32% of the responses, the criteria established in the drug´s Data Sheet are followed; in 26%, the consensus reports of the SEOM-SEAP (Spanish Society for Pathologic Anatomy) or the clinical guidelines are followed; in 20%, the guidelines of the IPTs are followed; and 12% follow reports of the corresponding Autonomous Community.

The determination of the biomarkers included in this study is outsourced to other centres in the following varying percentages: 29% for PDL-1, 36% for BRAF, 37% for ALK, 47% for ROS-1, and 63% for BRCA1/BRCA2. In the remaining cases, determinations are carried out in house.

### Biomarkers funding

Responses regarding the source of funding for each biomarker vary between AACC, as well as between hospitals within a given Autonomous Community and between different biomarkers within a given hospital.

In more than 50% of the participating centres, pharmaceutical companies are the source funding for the determination for clinical purposes of at least one of the biomarkers included in the study. In the remaining centers, the source of funding, public or private, cannot be determined due to the type of answers obtained.

A vast majority of the centres indicate that they do not employ any computer platform to manage and register the determination of predictive biomarkers. These kinds of registries are used when determinations are outsourced to other centres and are funded by pharmaceutical companies.

### Time taken from the request until results

The average estimated time taken from the request of the determination for the predictive biomarkers until the attainment of results varies between biomarkers, AACC and hospitals.

Median times vary from 7 days in PD-L1 in advanced NSCLC (from 2 to 21 days) to 29 days for BRCA1/BRCA2 in advanced ovarian cancer (from 1 to 240 days). The median time for BRAF in metastatic melanoma is 8 days (from 1 to 31 days); whilst in ALK and ROS1 in advanced NSCLC is of 10 days (from 2 to 30 days).

In centres which outsource these determinations, the time taken until obtaining a response is notably greater than in those where the determination is carried out in house. In some cases, these differences can be of up to 90 days.

### Barriers and limitations to the determination of the biomarkers

Amongst the primary barriers and/or limitations to the access to the biomarkers included in this study, participants have stated: the lack of standardized authorization procedures and of clear decision-making mechanisms; the dependency on the pharmaceutical industry for funding and, on occasions, for the designation of reference centres; in some cases, a lack of access to the determination; the time until the attainment of results, which is, in many cases, related to the outsourcing of the determination. In private hospitals, the primary barrier is the lack of coverage by private insurance companies.

## Discussion

The SEOM, from its commitment towards excellence in the care of cancer patients, carried out an initial study in 2015 to identify the difficulties and differences in the access to oncology drugs between hospitals and AACC in Spain [11]. With this present study, SEOM wanted to analyse the current situation, 4 years later, as well as to include in the study an analysis of the access to predictive biomarkers, essential tools for precision medicine. In fact, SEOM, together with the Spanish Society for Pathological Anatomy (SEAP) and the Spanish Society for Hospital Pharmacists (SEFH), has defended the need to develop a National Precision Medicine Strategy [12].

The SEOM acknowledges of the existing need for a health system planning based both on the quality of care as on equal access and still considers it a priority to identify inequalities and issue proposals for improvement with the aim of guaranteeing the accessibility of pharmaceutical drugs and biomarkers in equitable conditions throughout the national territory, without affecting the measures taken by AACC to rationalize use of drugs and health products, as established in the modification of Article 88.1 of December 2014 of Law 29/2006 on Guaranties and Rational Use of Drugs and Health Products.

The results show that there are significant differences between AACC regarding the scope of which decisions relevant to drug accessibility are made. Nevertheless, this analysis shows a tendency towards a greater centralization of decisions at an autonomic level. The responses indicate that in 51% of cases, decisions are made at a hospital/health área level (65.3% of cases in 2015), and that in 42% of cases, decisions are made at a regional level (27.8% of cases in 2015). In 58% of all responses, the participants indicate that the composition of commissions and other decision-making bodies is either not publically accessible or that there is no information available in this regard. When this information is available, participants indicate that such commissions include at least one medical oncologist, while 9.7% of hospitals indicated that no medical oncologist formed part of them in the study carried out in 2015.

The period to access to drugs of unquestionable therapeutic value is excessively long and, above all, there are significant differences at a regional level, and even at a hospital level within a given region. Without a doubt, this is a source of significant inequalities in the health system. Furthermore, 43% of the participant oncologists identify access barriers to the use of, at least, one of the drugs analyzed. In the study carried out in 2015, 37.8% of participants identified barriers or limitations for the access to the oncology drugs analyzed.

With regards to the access of biomarkers, in Spain, there is neither standardized procedure nor regulatory framework for the evaluation, implementation, and funding of biomarkers that predict the efficacy of specific oncology drugs in a given patient. These biomarkers are not included in the national portfolio of health services and, therefore, are authorised at regional or hospital levels following very different criteria and with varying approval timeframes. In fact, 84% of the centres included in this study do not have a commission or decision-making body responsible for managing the access of predictive biomarkers, or at least the participants have no information in this regard.

Other relevant areas of inequality include the funding of predictive biomarker determinations and the selection of hospital centres where such determinations are carried out. This study shows that pharmaceutical companies play a significant role in the funding, and, thus, in the organization of resources and the access to the biomarkers/indications that have been analyzed.

Concerning response times, the estimated time from the request for the determination of the biomarkers/indications until the availability of a result varies significantly between biomarkers, AACC, and hospitals. The time taken is notably greater in centres that outsource the determination than where the determination is carried out in house.

The differences in the access to oncology drugs and predictive biomarkers could impact on the health outcomes, even though this impact has not been measured in this study due to the limitations in its scope.

Given this situation, the SEOM expresses its concern regarding the variability in the access to oncology drugs as well as to biomarkers which are necessary for the correct selection of certain treatments that have an unquestionable clinical benefit, in the different AACC and in different hospital centres within the same Autonomous Community.

The SEOM, therefore, considers it necessary to implement initiatives with the following objectives:

1. Include biomarkers in the portfolio of common services of the Spanish National Health System (SNS).
2. Develop a standardized procedure and a regulatory framework for the implementation and funding of the biomarkers.
3. Determine which biomarkers should be carried out in house and which must be carried out in reference centres.
4. Reduce the identified differences and increase homogeneity in the access to drugs in Spain, demanding that the same conditions apply throughout the Spanish territory and that the competent decision-making bodies approve these common conditions.
  To achieve this, there is a need to:
  Ensure total transparency in the composition and decisions of commissions and other decision-making bodies
  Eliminate barriers at a regional level (AACC) and hospital level
5. Reduce existing delays between the date of approval of drugs for their commercialization in Spain and the date of approval for their prescription to patients.
  To achieve this, there is a need to:
  Limit the time taken for the inclusion of new drugs in the AACC following the authorization by the Spanish Ministry of Health.
  Eliminate bureaucratic procedures, such as additional supporting reports following the attainment of published IPTs as well as Scientific Society reports prepared by experts.

Measure health outcomes to understand the real impact of introducing a new drug, using the tools designed in collaboration with the General Directorate of Basic Portfolio of Services of the Spanish National Health System and Pharmacy, and that these be used in every Autonomous Community with the same criteria.

## Conclusions

This study shows that there are still variations in the access of drugs as well as of biomarkers that predict the efficacy of different oncology drugs between different AACC as well as at a hospital level within a given Autonomous Community.

In addition to the significant differences in the decision-making process, access times for these drugs are still worrisome, not only due to its length, but also due to the notable differences between AACC and between hospitals for a given drug. Additionally, the fact that biomarkers are not included in the basic portfolio of services of the SNS means that biomarkers are introduced differently in the different AACC and in different hospitals.

This situation could directly affect patients, whose access to certain drugs and predictive biomarkers depends on the region and on the centre where they are being treated.

For this reason, the SEOM expresses its concern and considers it necessary to implement initiatives to (a) reduce the detected differences to ensure equity in drug access, regardless of the patients´ place of residence, and reduce delays in the time taken from the EC authorization until use in hospitals, (b) include biomarkers in the basic portfolio of drugs and services of the SNS, and (c) measure health outcomes to understand the real impact of introducing new treatments and biomarkers.

The SEOM would like to offer itself as a collaborative partner for the Spanish health authorities in promoting initiatives aimed at ensuring equity and the optimal access of oncology drugs and biomarkers, regardless of a patients´ place of residence.

## Appendix

### Appendix 1. Descriptive data requested for each centre

1. Select your province (MANDATORY)
2. Select the level of your hospital
3. Select the number of inhabitants of your reference population
4. Select the scope of influence of the Pharmacotherapy Commission where binding decisions regarding the drug access in your centre are made: (Please select one option)
  - ☐ Hospital
  - ☐ Group of hospitals
  - ☐ Health area
  - ☐ Province
  - ☐ Autonomous Community
  - ☐ Unknown
  Do you know the number of members that form the previously mentioned Commission?
  - ☐ Yes
  - ☐ No
  If the chosen answer is “YES”, please indicate the following:
  Number of members in the previously mentioned Commission:
  Number of medical oncologists that take part in the commission:
  Is this information publically available?
  - ☐ Yes
  - ☐ No ☐Unknown
5. Are there any electronic prescription mechanisms available? Please indicate.
6. Select the level that decisions regarding the determination of predictive biomarkers of response to treatment with oncology drugs are made:
  - ☐ Hospital
  - ☐ Group of hospitals
  - ☐ Health area
  - ☐ Province
  - ☐ Autonomous Community
  - ☐ Unknown
  - ☐ No binding decisions are made at any level
7. In your centre, is there a Commission or any other decision-making body responsible for making decisions regarding the access to predictive biomarkers of response to treatment with oncology drugs?
  - ☐ Yes (please indicate which)
  - ☐ No ☐ Unknown
  If the selected answer is “YES”, is the information on its existence and means of operation publically available?
8. Additional comments

### Appendix 2. Requested data on the access of the drugs and indications analysed (for each drug/indication)

1. Has the request for the use of the drug in your centre been presented to the Pharmacotherapy Commission?
  - ☐ Yes
  - ☐ No
  - ☐ No, approval is granted by the regional Commission where presenting the drug is not necessary
  - ☐ Unknown
    If the answer is “No”, what was the primary motive for not presenting a request?
    - ☐ Unofficial negative response
    - ☐ Excessive delay
    - ☐ Excessive burocracy
    - ☐ It does not affect my prescription
2. Has the drug been approved for clinical use for the given indication in your hospital?
  - Yes ☐
  - No ☐
  - Pending evaluation ☐ (indicate date)
    If the chosen answer is “YES”, please indicate the date of approval (month/year)
    If the chosen answer is “NO”, have patients been derived to other centres that have access to the drug?
    If the chosen answer is “NO”, are there any alternative prescription mechanisms in place that grant access to the drug?
    Yes ☐ (Please indicate) No ☐
3. If the use of the drug has been approved in the hospital for the given indication, is its use governed by/dependent on the criteria established in…? Please select one or more of the following options:
  - ☐ Data Sheet (Ficha Técnica, FT)
  - ☐ It has been approved under the same conditions as the Data Sheet (FT)
  - ☐ It has been approved under more restrictive conditions than those of the Data sheet (FT)
  - ☐ It has been approved under less restrictive conditions than those of the Data Sheet (FT)
  - ☐ Therapeutic Positioning Report (Informe de Posicionamiento Terapéutico, IPT)
  - ☐ It has been approved under the same conditions as the Therapeutic Positioning Report (IPT)
  - ☐ It has been approved under more restrictive conditions than those of the Therapeutic Positioning Report (IPT)
  - ☐ It has been approved under less restrictive conditions than those of the Therapeutic Positioning Report (IPT)
  - ☐ Regional Report (Autonomous Community)
  - ☐ Hospital Report prepared by the Pharmacotherapy Commission
  - ☐ SEOM Evaluation Report
  - ☐ Genesis Evaluation Report
  - ☐ The drug is not available in the centre
  - ☐ Others Please specify
4. Can you identify any barriers / limitations to the access of the drug for the given indication in your center?:
  No ☐ Yes ☐
  If the chosen answer is yes, please select one or more of the following options regarding the barriers to access the drug in your centre:
  - ☐ The requirements established by the centre are more restrictive than those included in the Data Sheet
  - ☐ Drafting of a supporting report specific to each patient is required
  - ☐ Expenditure capping systems have been implemented
  - ☐ No medical oncologists take part in the Pharmacotherapy Commission
  - ☐ Lack of hospital budget
  - ☐ A different drug with the same indication but a lower price has been approved
  - ☐ The sequence of treatments for the same indication are not permitted
  - ☐ Other barriers to the use. Please specify which
5. Please add any other relevant comments.

### Appendix 3. Requested data on the access to the analysed biomarkers (for each biomarker/indication)

1. In your centre, is it possible to request the determination of the biomarker as a predictive biomarker of response to the treatment of the given indication?
  - ☐ Yes
  - ☐ No
  1.1 If the chosen answer is “YES”, since when has the use of the biomarker been possible (month and year)?
    Multiple choice:
  1.2 If the chosen answer is “NO”, what is the reason for its inaccessibility?
  Multiple choice:
  - ☐ Negative response from the decision-making bodies
  - ☐ Delay in the response
  - ☐ Procedures for presenting the request are unknown/inexistent
  - ☐ Other motives. Please specify which. Click here to type text.
2. Is the use of the biomarker as a predictive biomarker of response to treatment for a different type of cancer included in the hospital´s portfolio of available services?
  - ☐ Yes. Please specify
  - ☐ No.
3. What are the available techniques for the determination of the biomarker as a predictive biomarker in response to the treatment of the given indication?
4. Who is responsible for carrying out the *determination* of the biomarker as a predictive biomarker in response to the treatment of the given *indication*?
  The hospital itself ☐ Outsourced ☐
  Do other centres outsource their determinations to your centre?
  - ☐ Yes. Please specify which centres. Click here to type text.
  - ☐ No.
  - ☐ Unknown.
    If determinations are outsourced, please specify to which centre
5. Is the use of the *biomarker* as a predictive biomarker in response to the treatment of the given *indication* governed by/ dependant on the criteria established in…? Please select one or more of the following options:
  - ☐ Therapeutic Positioning Report (IPT) Please specify which drug
  - ☐ Data Sheet (FT) Please specify which drug
  - ☐ Regional Report (Autonomous Community)
  - ☐ Hospital Report
  - ☐ Clinical Guidelines / SEOM Consensus Reports
  - ☐ Unknown
  - ☐ The biomarker is not available in the centre
  - ☐ Other Please specify
6. Who is responsible for funding the *biomarker* determination for the purpose of predictive care for the *indication* in your hospital?
  - ☐ Regional Health Services / Regional Ministries
  - ☐ The Hospital
  - ☐ Pharmaceutical Industry
  - ☐ Research funds
  - ☐ Other. Please specify
7. Do you use any IT platform to manage and register the determination of a *biomarker* as a predictive *biomarker* in response to the treatment of a given *indication?*
  - ☐ Yes, please specify
  - ☐ No
8. What is the average response time from the application for the determination until de availability of the result?
9. Can you identify any barriers/limitations in your centre to the access of the *biomarker* as a predictive biomarker in response to the treatment of the given *indication*?
  Yes ☐ please specify No ☐
10. Please add any other relevant comments.
